# Availability and affordability of new medicines in Latin American countries where pivotal clinical trials were conducted

**DOI:** 10.2471/BLT.14.151290

**Published:** 2015-07-29

**Authors:** Núria Homedes, Antonio Ugalde

**Affiliations:** a Houston Health Science Center, School of Public Health, El Paso Regional Campus, University of Texas, 1101 North Campbell Street, El Paso 79902, United States of America (USA).; b Department of Sociology, University of Texas at Austin, Texas, USA.

## Abstract

**Objective:**

To assess whether new pharmaceutical products approved by the United States Food and Drug Administration (FDA) in 2011 and 2012 were registered, commercialized and sold at affordable prices in the Latin American countries where they were tested.

**Methods:**

We obtained a list of new molecular entities (new pharmaceutical products) approved by the FDA in 2011 and 2012. FDA medical reviews indicated the countries where pivotal clinical trials had been conducted. The registration status of the products was obtained from pharmaceutical registers; pharmaceutical companies confirmed their availability in national markets and local pricing observatories provided the price of medicines in retail pharmacies. Affordability was assessed as the cost of a course of treatment as a proportion of monthly income. Information on safety and efficacy was gathered from independent drug bulletins.

**Findings:**

Of an expected 114 registrations, if the 33 products had been registered in all the countries where tested, only 68 (60%) were completed. Eight products were registered and commercialized in all countries but 10 had not been registered in any of the countries. With one exception, products for which we obtained pricing information (*n* = 18) cost more than the monthly minimum wage in all countries and 12 products cost at least five times the monthly minimum wage.

**Conclusion:**

Many pharmaceutical products tested in Latin America are unavailable and/or unaffordable to most of the population. Ethical review committees should consider the local affordability and therapeutic relevance of new products as additional criteria for the approval of clinical trials. Finally, clinical trials have opportunity costs that need to be assessed.

## Résumé

**Objectif:**

Évaluer si les nouveaux produits pharmaceutiques approuvés par la *Food and Drug Administration* (FDA) des États-Unis en 2011 et 2012 ont été homologués, mis sur le marché et vendus à des prix abordables dans les pays d'Amérique latine où ils ont été testés.

**Méthodes:**

Nous nous sommes procuré la liste des nouvelles entités moléculaires (nouveaux produits pharmaceutiques) approuvées par la FDA en 2011 et 2012. Les comptes-rendus médicaux de la FDA indiquaient les pays dans lesquels des essais cliniques pivots avaient été menés. Le statut des produits en matière d'homologation nous a été révélé par les registres pharmaceutiques; les sociétés pharmaceutiques ont confirmé la disponibilité des produits sur les marchés nationaux et les observatoires locaux des prix nous ont communiqué le prix des médicaments dans les pharmacies de détail. Leur accessibilité financière a été évaluée au regard du revenu mensuel. Des informations sur leur innocuité et leur efficacité ont été recueillies dans des bulletins pharmaceutiques indépendants.

**Résultats:**

Sur les 114 homologations escomptées si les 33 produits avaient été homologués dans tous les pays où ils ont été testés, seules 68 (60%) étaient accordées. Huit produits étaient homologués et commercialisés dans tous les pays, mais 10 n'avaient été homologués dans aucun des pays. À une exception près, les produits pour lesquels nous avons obtenu des informations tarifaires (*n* = 18) ont un prix supérieur au salaire minimum mensuel dans tous les pays et 12 produits coûtent au moins cinq fois plus que le salaire minimum mensuel.

**Conclusion:**

De nombreux produits pharmaceutiques testés en Amérique latine sont indisponibles et/ou inaccessibles financièrement pour la majeure partie de la population. Les comités d'éthique devraient envisager de prendre en compte l'accessibilité financière et l'intérêt thérapeutique des nouveaux produits au niveau local comme critères supplémentaires d'approbation des essais cliniques. Enfin, les essais cliniques ont des coûts d'opportunité qui doivent être évalués.

## Resumen

**Objetivo:**

Evaluar si los nuevos productos farmacéuticos aprobados por la Administración de Alimentos y Medicamentos (FDA) de los Estados Unidos en 2011 y 2012 fueron registrados, comercializados y vendidos a precios asequibles en los países de América Latina en los que se probaron.

**Métodos:**

Se obtuvo una lista de las nuevas entidades moleculares (los nuevos productos farmacéuticos) aprobadas por la FDA en 2011 y 2012. Los análisis médicos de la FDA indicaron los países en los que se habían llevado a cabo los ensayos clínicos decisivos. El estado del registro de los productos se obtuvo de los registros farmacéuticos; las empresas farmacéuticas confirmaron su disponibilidad en los mercados nacionales y los observatorios de precios proporcionaron el precio de los medicamentos en farmacias minoristas. La asequibilidad se evaluó como el costo de un ciclo de tratamiento en proporción a los ingresos mensuales. La información sobre seguridad y eficacia se obtuvo de boletines independientes de medicamentos.

**Resultados:**

De los 114 registros esperados si los 33 productos se hubieran registrado en todos los países en los que se habían probado, solo se completaron 68 (el 60%). Se registraron y comercializaron ocho productos en todos los países, pero 10 no habían sido registrados en ninguno de los países. Con una excepción, los productos sobre los que obtuvimos información sobre precios (*n* = 18) costaban más que el sueldo mínimo mensual en todos los países y 12 productos costaban al menos cinco veces el sueldo mínimo mensual.

**Conclusión:**

Varios productos farmacéuticos probados en América Latina no se encuentran disponibles o no son asequibles para la mayor parte de la población. Los comités de revisión ética deberían considerar la asequibilidad local y la relevancia terapéutica de los nuevos productos como un criterio adicional a la hora de aprobar ensayos clínicos. Finalmente, los ensayos clínicos tienen costes de oportunidad que necesitan ser evaluados.

## ملخص

**الغرض:**

إجراء تقييم ****للتأكد من أن المنتجات الدوائية الجديدة المعتمدة من إدارة الأغذية والعقاقير بالولايات المتحدة الأمريكية (FDA) في عامي 2011 و2012، قد تم تسجيلها واستخدامها تجارياً وبيعها بأسعار ميسورة في دول أمريكا اللاتينية حيث تم اختبارها.

**الطريقة:**

حصلنا على قائمة بالكيانات الجزيئية (المنتجات الدوائية الجديدة) المعتمدة من إدارة الأغذية والعقاقير في عامي 2011 و2012. وأوضحت المراجعات الطبية لإدارة الأغذية والعقاقير الدول التي تم إجراء التجارب السريرية المحورية بها. كما تم الحصول على حالة تسجيل هذه المنتجات من القائمين على التسجيل الدوائي؛ وأكدت شركات الأدوية توافر هذه المنتجات في الأسواق المحلية، كما أفادت الجهات المحلية المعنية بمراقبة الأسعار، بأسعار الأدوية في الصيدليات التي تتولى بيعها بالتجزئة. وتم تقييم معقولية الأسعار على أنها تكلفة دورة من العلاج باعتبارها نسبة من الدخل الشهري. وتم جمع بعض المعلومات عن سلامة وكفاءة المنتجات من خلال النشرات المستقلة المتعلقة بالعقاقير.

**النتائج:**

إذا ما تم تسجيل 33 منتجاً في كل الدول التي تم اختبار المنتجات بها، وذلك من بين 114 عملية تسجيل متوقعة، فإن عمليات التسجيل المكتملة تبلغ 68 عملية (60%) فقط. وتم تسجيل ثمانية منتجات واستخدامها تجاريًا في كافة الدول، ولكن لم يتم تسجيل 10 منها في أية دولة. وبلغت تكلفة المنتجات التي حصلنا على معلومات الأسعار بشأنها (العدد = 18) ما يزيد على الحد الأدنى للراتب الشهري في جميع الدول، بينما بلغت تكلفة 12 منتجاً ما يساوي خمس أضعاف الحد الأدنى للراتب الشهري، وذلك باستثناء منتج وحيد.

**الاستنتاج:**

لا يتوافر الكثير من المنتجات الدوائية التي تم اختبارها في أمريكا اللاتينية و/أو لا يمكن الحصول عليها بأسعار معقولة لمعظم سكان هذه المنطقة. ويجب على لجان المراجعة الأخلاقية النظر في مدى معقولية الأسعار على المستوى المحلي والملاءمة العلاجية للمنتجات الجديدة باعتبار ذلك معيار إضافي لاعتماد التجارب السريرية. وأخيرًا، تحتاج تكاليف فرص التجارب السريرية إلى التقييم.

## Abstract (es)

进行了关键临床试验的拉丁美洲国家对新药品的可得性和负担能力

## 摘要

**目的:**

评估美国食品药物管理局 (FDA) 2011 年和 2012 年批准的、在拉丁美洲国家进行试验的新药品是否已注册且商品化，是否以负担得起的价格出售。

**方法:**

我们获得了 (FDA) 2011 年和 2012 年批准的新分子实体药物（新药品）的清单。(FDA) 医疗审查表明进行关键临床试验的国家。 药品的注册状态从药品注册处获得，制药公司在全国市场确定药品的可得性，当地价格监测站提供药品在零售药房的价格。 负担能力按照一个疗程的费用在月收入中所占比例进行评估。 安全和疗效信息从独立的药物公告收集。

**结果:**

如果 33 种药品已经在试验的所有国家注册过，在预期的 114 个注册国家中，只有 68 个 (60%)  已完成。 8 种药品在所有国家注册过和商品化，但有 10 种药品还未在任何国家注册过。 从我们获得的价格信息 (*n* = 18) 来看，除了一种例外，所有国家的药品价格都超过了月最低收入，有 12 种药品的价格至少是月最低收入的五倍。

**结论:**

许多在拉丁美洲试验的药物都不可得且/或大部分人负担不起。 伦理审查委员会应考虑新药品的当地负担能力和治疗相关性，以此作为批准临床试验的附加标准。 最后，临床试验有需要进行评估的机会成本。

## Резюме

**Цель:**

Оценка того, были ли зарегистрированы, выпущены в продажу и доступны по приемлемым ценам новые виды фармацевтической продукции, одобренные Управлением США по контролю качества пищевых продуктов и лекарственных средств (FDA) в 2011 и 2012 гг., в тех странах Латинской Америки, в которых проводились их испытания.

**Методы:**

Мы получили список новых молекулярных единиц (новой фармацевтической продукции), одобренных FDA в 2011 и 2012 гг. В медицинских обзорах FDA указывались страны, в которых проводились опорные клинические исследования. Сведения о регистрации продукции были получены из фармацевтических реестров. Фармацевтические компании подтвердили наличие продукции на национальных рынках, а местные агентства по контролю за ценами предоставили данные о стоимости тех или иных лекарств в розничной продаже (в аптеках). Ценовая доступность продукции основывалась на том, какую долю стоимость лекарств занимает в ежемесячном доходе. Независимые бюллетени лекарственных средств предоставили информацию о безопасности и эффективности препаратов.

**Результаты:**

Из ожидаемых 114 регистраций по результатам испытания 33 видов продукции во всех странах-участницах испытаний фактически было зарегистрировано только 68 препаратов (60%). Восемь видов продукции были зарегистрированы и выпущены в продажу во всех странах. Десять препаратов не имели регистрации ни в одной из стран. За одним исключением, продукция, для которой удалось получить информацию о ценах (*n* = 18), стоила выше минимального ежемесячного дохода во всех странах, а 12 видов продукции стоили по меньшей мере впятеро больше минимального ежемесячного дохода.

**Вывод:**

Многие виды фармацевтической продукции, проходившей испытания в странах Латинской Америки, либо отсутствуют на рынках этих стран, либо имеются, но недоступны большинству населения из-за цены. Комитеты по этике должны учитывать доступность препаратов на местах и терапевтическую релевантность новой продукции в качестве дополнительных критериев при одобрении проведения клинических исследований. Кроме того, с клиническими исследованиями сопряжены альтернативные издержки, которые также требуют оценки.

## Introduction

The high cost of many new medicines calls into question whether people in low- and middle-income countries will have access to them,^1^ yet an increasing number of pivotal trials are carried out in these countries. Pivotal clinical trials are those included in the applications for market authorization or new drug approval documents submitted to regulatory agencies.

This paper explores two issues: (i) are new molecular entities (hereafter products) approved by the FDA in 2011 and 2012 available in Latin American countries where the pivotal trials were conducted? and (ii) if registered, are they marketed at affordable prices? We also obtained information about the therapeutic relevance of the new products from the databases of two independent drug bulletins.

## Methods

This is a cross-sectional study. We obtained the list of new products approved by the United States Food and Drug Administration (FDA) in 2011 and 2012.^2^^,^^3^ One product, Gadobutrol (Gadovist), was approved during the study period but excluded from the study because it is a contrast dye used in radiology, not a pharmaceutical treatment. The FDA’s medical reviews of the new products, included in their drug approval history,^4^ provided the names of countries where the trials had been conducted. If this information was not available in the medical reviews, we obtained it from the trial sponsors.

### Obtaining regulatory status

We searched the pharmaceutical registers for the regulatory status of relevant products in each country. The information included in the registers varies slightly by country. Brazil, Chile and Colombia maintain a register of approved pharmaceuticals; Argentina has a register of marketed products; Mexico publishes a list of the products approved per time period and Peru catalogues products available in pharmacies (See Box 1 for a list of web sites consulted). For the countries without registers (Costa Rica, Ecuador, Panama, Peru, Uruguay) we approached the regulatory agencies. We were unable to reach the regulators in the Dominican Republic or the Bolivarian Republic of Venezuela.

Box 1 **Databases consulted to obtain registration status of drugs tested and the price of pharmaceuticals**

**Argentina**

- Vademecum Nacional de Medicamentos, ANMAT^5^
- Listado Oficial de Medicamentos Comercializados LOMAC, ANMAT^6^

**Brazil**

- Drug price list, Agência Nacional de Vigilância Sanitária^7^
- Medications that have been analysed, Agência Nacional de Vigilância Sanitária^8^

**Chile**

- Query system of registered products, Instituto de Salud Pública^12^
- Pharmaceucial prices, Precio de Remedios^13^

**Colombia**

- Product data - Instituto Nacional de Vigilancia de Medicamentos y Alimentos (INVIMA)^9^
- List of chemical entities with unpublished information protected by Decree 2085 of 2002 - Instituto Nacional de Vigilancia de Medicamentos y Alimentos (INVIMA)^10^
- Drug price – Circular 2, 2012, Ministerio de Salud y Protección Social^11^

**Mexico**

- Price list, Ministry of Economy^14^
- Pharmaceutical prices, Precio de Remedios^15^

**Peru**

- Catálogo de Productos Farmacéuticos, Ministerio de Salud^16^Módulo de Consulta de Precios, Ministerio de Salud^17^

We contacted pharmaceutical companies’ headquarters in the United States of America to gather information on the marketing status of their products in the selected countries.

### Cost of medicines

In Latin America, about 78% of all medicines are paid for out-of-pocket in retail pharmacies.^18^ Since the products of interest were not included in the World Health Organization (WHO)/Health Action International (HAI) medicine prices’ database, we obtained the price of the unit dose of each product from the countries’ price observatories, which report the maximum price to consumers (Brazil, Mexico) or the observed consumer prices (Argentina, Chile, Colombia, Ecuador, Peru; Box 1).

The consumer prices for Argentinean products not tracked by its observatory and for products in Costa Rica, where there is no observatory, were provided by pharmacological experts who obtained them from local distributors. The quantities needed to complete a course or a year of treatment were calculated using the FDA-approved product label. The pricing information was gathered between 25 August and 20 September, 2014.

### Commercialization status

Obtaining information on the commercialization status of each product from the pharmaceutical companies was difficult. The headquarters of some companies responded quickly with the requested information (Vertex, Exelixis, Glaxo Smith Kline (GSK), Bristol-Myers Squibb (BMS), Sanofi, AstraZeneca), while others referred us to their country subsidiaries or to the companies responsible for commercializing their products outside the United States of America (USA). The accuracy of the information provided depended on the familiarity of the respondent with the company’s practices and databases. Two companies provided contradictory information and one referred us back and forth between the original producer and the licensee. With few exceptions (Pfizer Brazil, Colombia and Mexico; Janssen Argentina; Novartis Argentina and Colombia; Takeda Brazil and Boehringer Mexico), the Latin American offices were less willing to share information than the respondents at headquarters. One company representative wrote: “In response to your question below, we have a policy of restricting the disclosure of proprietary business information/strategies unless we have a formal business relationship protected by a confidential disclosure agreement.”

Information on registration status and pricing helped resolve some of the inaccuracies of information reported by industry. For example, pricing information indicated the probable availability of pertuzumab in Mexico, rivaroxavan in Colombia and Mexico and ticagrelor in Argentina. We decided that bosutinib was not marketed in Argentina or Peru since it was not included on the list of marketed products (Argentina) or in the catalogue of products available in pharmacies (Peru) and because price information was not available in either country.

It was impossible to confirm the marketing status in 10 cases (pasireotide in Brazil; rilpivirine in Argentina, Chile and Mexico; pertuzumab in Peru; teriflunomide in Chile and Mexico; tofacitinib in Costa Rica and Peru; and vandetanib in Mexico).

### Measuring affordability

There are challenges in gathering information related to the price of medicines and incomes to determine affordability thresholds.^19^ Some argue that a threshold of 5% of total expenditure for the purchase of medicines would classify them as unaffordable in countries such as India and Indonesia.^20^ Some authors consider health-care expenditure catastrophic if it exceeds 10% of yearly household income.^21^ In this study, the affordability of each course of treatment is presented in relation to the monthly household or per capita income. Whether or not this is a catastrophic expenditure depends on the socioeconomic status of the individual or the household. In regions with large income inequalities, such as Latin America, using population averages can be misleading.

Wealth was measured using: (i) monthly minimum wages in 2014, obtained from public announcements in the media; (ii) average monthly per capita income, from a World Bank database; (iii) the monthly household net adjusted disposable income, and (iv) the monthly household financial wealth which was only available for Brazil, Chile and Mexico (Table 1).

**Table 1:** Monthly minimum wage, monthly income per capita, household net-adjusted disposable income and household financial wealth

Country	Monthly minimum wage,^a^ US$	Mean monthly income per capita,^b^ US$	Average monthly household net-adjusted disposable income,^c^ US$	Average monthly household financial wealth,^d^ US$
Argentina	523 (since 9/2014)	1230	NA	NA
Brazil	329 (since 7/2014)	929	859	573
Chile	387 (since7/2014)	1311	1147	1512
Colombia	306 (since 1/2014)	652	NA	NA
Ecuador	340 (since 1/2014)	477	NA	NA
Mexico	111 (since 1/2014)	859	1071	871
Peru	259 (since 6/2012)	550	NA	NA

NA: not available; OECD: Organisation for Economic Co-operation and Development; US$: United States dollars.

^a^ The minimum wage per country was obtained from public announcements in the media. Local currencies were exchanged into US$ according to the official exchange rate of 1 September 2014.

^b^ Income per capita from the 2013 database of the World Bank adjusted by authors to the mean value.

^c^ Household net-adjusted disposable income from the *OECD Better life index 2014*. It is defined as the amount of money that a household earns, or gains, each year after taxes and transfers. It represents the money available to a household for spending on goods or services. The income reported in this publication is for 2011, http://www.oecdbetterlifeindex.org/topics/income/.

^d^ Household financial wealth is from the *OECD Better life index 2014*. It is defined as the total value of a household’s financial worth, or the sum of its overall financial assets minus liabilities. Financial wealth takes into account: savings, monetary gold, currency and deposits, stocks, securities and loans. The wealth reported in this publication is for 2011, http://www.oecdbetterlifeindex.org/topics/income/.

Finally, we consulted the databases of two independent drug bulletins (Prescrire, France [http://www.prescrire.org/fr/Search.aspx], and the Health Research Group – Public Citizen, USA [http://www.worstpills.org/search/]) for assessments of the clinical relevance of the new products compared with existing treatments.

## Findings

The 33 products included in this study are shown in Table 2. Of an expected 114 registrations, if the 33 products had been registered in all the countries where tested, 60% (68/114) were completed. Three cases were excluded as the regulatory status of the product could not be determined by 20 September 2014.

**Table 2:** Products approved by the United States Food and Drug Administration in 2011 and 2012 that were tested in pivotal trials in Latin America

Non-proprietary name	Commercial name	Pharmaceutical company	Countries where pivotal clinical trials were conducted
Aclidium bromide	Tudorza Pressair®/Eklaire Genuari®	Forest/Almirall	Peru
Aflibercept	Eylea®/Eylia®	Bayer	Argentina, Brazil, Chile, Colombia, Mexico
Apixaban	Eliquis®/Elicuis®	BMS	Argentina, Brazil, Chile, Colombia, Mexico, Peru
Axitinib	Inlyta®	Pfizer	Brazil
Azilsartan, medoxomil	Edarbi®	Takeda	Argentina, Chile, Mexico, Peru
Bedaquiline	Sirturo®	Janssen	Brazil
Belatacept	Nulojix®	BMS	Argentina, Brazil, Chile, Mexico,
Belimumab	Benlysta®	GSK	Argentina, Brazil, Chile, Colombia, Costa Rica, Mexico, Peru
Bosutinib	Bosulif®	Pfizer	Argentina, Brazil, Chile, Colombia, Mexico, Peru
Cabozantinib	Cometriq®	Exelixis/Sobi	Brazil, Chile, Peru
Crizotinib	Xalkori®	Pfizer	Brazil
Elvitegravir/obicistat/ emtricitabine/tenofovir disoproxil fumarate	Stribild®	Gilead	Mexico
Enzalutamide	Xtandi®	Raffo/Astellas	Argentina, Chile
Ezogabine	Potiga®	GSK	Argentina, Brazil, Mexico
Indacaterol maleate	Arcapta Neohaler®/Onbrize®	Novartis	Argentina, Chile, Colombia, Ecuador, Peru
Ipilimumab	Yerboy®/Yervoy®	BMS	Argentina, Brazil, Chile, Peru
Linagliptin	Tradjenta®	Boehringer	Argentina, Mexico
Lucinactant	Surfaxin®	Discovery	Brazil, Chile, Ecuador, Mexico, Panama, Uruguay
Pasireotide	Signifor®	Novartis	Argentina, Brazil, Mexico
Perampanel	Fycompa®	Eisai	Argentina, Chile, Mexico
Pertuzumab	Perjeta®	Genentech/Roche	Brazil, Mexico, Peru
Regorafenib	Stivarga®	Bayer	Argentina, Brazil
Rilpivirine	Edurant®	Janssen	Argentina, Brazil, Chile, Costa Rica, Mexico, Panama
Rivaroxavan	Xarelto®	Bayer/Janssen	Argentina, Brazil, Chile, Colombia, Mexico, Peru, Venezuela (Bolivarian Republic of)
Roflumilast	Daliresp®/Daxas®	Forest/Takeda	Brazil
Taliglucerase alfa	Elelyso®/Uplyso®	Pfizer	Chile
Tbo-filgastrim	Neutroval®/Granix®	Teva	Brazil, Chile
Telaprevir	Incivek®	Janssen/Vertex	Argentina, Brazil
Teriflunomide	Aubagio®	Genzyme	Chile, Colombia
Ticagrelor	Brilinta®	AstraZeneca	Argentina, Brazil, Mexico,
Tofacitinib	Xeljan®	Pfizer	Argentina, Brazil, Chile, Colombia, Costa Rica, Dominican Republic, Mexico, Peru, Venezuela (Bolivarian Republic of)
Vandetanib	Caprelsa®	AstraZeneca	Argentina, Brazil, Mexico
Ziv-aflibercept	Zaltrap®	Sanofi	Argentina, Brazil, Chile, Colombia, Mexico

Combining information on registration and availability indicated that 30% (10/33) of products were not registered or commercialized in any of the countries where they had been tested (aclidium bromide, axitinib, bedaquiline, bosutinib, cabozantinib, elvitegravir/obicistat/emtricitabine/tenofovir disoproxil fumarate, lucinactant, perampanel, tbo-filgastrim, ziv-aflibercept); two products were registered but not marketed in any of the countries where tested (enzalutamide and ezogabine) and eight were registered and commercialized in all countries where tested (aflibercept, indacaterol maleate, ipilimumab, linagliptin, regorafenib, roflumilast, taliglucerase alfa, telaprevir).

Table 3 presents the approval and commercialization status in September 2014 of the products included in the study that were not registered or commercialized in all the countries where tested. In two cases the pharmaceutical company stated that a product was available when the regulatory agency said it had not been approved and in several cases the products were registered but not marketed. In total, by September 2014, 36.4% of products were not marketed in any of the countries where they were tested and only 51% were available where tested.

**Table 3:** Approval and marketing status in September 2014 of products tested in Latin American countries and approved by the FDA in 2011 and 2012^a^

Non-proprietary name	Argentina			Brazil			Chile			Colombia			Costa Rica			Mexico			Panama			Peru	
	R	M		R	M		R	M		R	M		R	M		R	M		R	M		R	M
Apixaban	Yes	A		Yes	A		Yes	A		Yes	A		–	–		Yes	NA^b^		–	–		Yes	NA
Azilsartan medoxomil	Yes	A		–	–		No	NA		–	–		–	–		Yes	A		–	–		No	NA
Belatacept	Yes	A		Yes	NA		No	NA		–	–		–	–		Yes	NA		–	–		–	–
Belimumab	Yes	A		Yes	A		Yes	A		No	NA		No	NA		Yes	A		–	–		No	A
Crizotinib	–	–		Yes	NA		–	–		–	–		–	–		–	–		–	–		–	–
Enzalutamide	Yes	NA		–	–		Yes	NA		–	–		–	–		–	–		–	–		–	–
Ezogabine	Yes	NA		No	NA		–	–		–	–		–	–		No	NA		–	–		–	–
Pasireotide	Yes	A		No	FA		–	–		–	–		–	–		Yes	A		–	–		–	–
Pertuzumab	–	–		Yes	A		–	–		–	–		–	–		Yes	A		–	–		Yes	FA
Rilpivirine	–	FA		No	NA		No	FA		–	–		No	NA		Yes	FA		Yes	NA		–	–
Rivaroxavan	Yes	A		Yes	A		Yes	A		Yes	A		–	–		Yes	A		–	–		Yes	A
Teriflunomide	–	–		–	–		Yes	FA		–	–		–	–		Yes	FA		–	–		–	–
Ticagrelor	Yes	A		Yes	FA		–	–		–	–		–	–		Yes	A		–	–		–	–
Tofacitinib	Yes	A		No	NA		Yes	A		Yes	A		No	C^c^		Yes	A		–	–		Yes	C
Vandetanib	Yes	A		Yes	A		–	–		–	–		–	–		No	FA		–	–		–	–

A: available; C: received contradictory information; FA: failed to get an answer; FDA: (United States) Food and Drug Administration; M: marketing status; NA: not available; R: registered.

^a^ The table does not include the eight new products that have been registered and commercialized in all the countries; neither does it include the ten new products that have not been registered or commercialized in any country.

^b^ BMS said that apixaban was not marketed in Mexico but we found its price. It may only be available for compassionate use.

^c^ The Caja Costarricense de Seguridad Social, the social security agency that provides health care including free medications to 90% of the Costa Rica’s population, has not bought tofacitinib and it is not available in major pharmacies.

Note: For cells with no information, no clinical trials with this product were conducted in the country.

The cost of one course of treatment (or a year of treatment for chronic conditions) is shown in Table 4. The prices of 18 of the 21 products marketed in Latin American countries for which we were able to obtain information varied widely. In all countries, and for all except one of the products, the cost was greater than the monthly minimum wage. Five products cost one to four times the monthly minimum wage, while six cost between 100 and 896 times this amount.

**Table 4:** Cost of a course of treatment (or a year of treatment for chronic conditions) for new products tested in Latin America in relation to the monthly minimum wage, 2014

Cost^a^	Argentina	Brazil	Chile	Colombia	Ecuador	Mexico	Peru
< 1	Rivaroxavan	Rivaroxavan	Rivaroxavan	Rivaroxavan	–	Rivaroxavan	Rivaroxavan
1–4	Apixaban, Indacaterol, Linagliptin, Ticagrelor	Apixaban, Roflumilast, Ticagrelor	Apixaban, Indacaterol	Apixaban, Indacaterol	Indacaterol	–	–
5–9	–	Belatacept	–	–	–	Azilsartan, Linagliptin	–
10–19	–	–	Belimumab	–	–	Apixaban, Ticagrelor	–
20–39	Aflibercept, Regorafenib	Aflibercept	Aflibercept	Aflibercept	–	–	–
40–59	Belatacept, Tofacitinib	Belimumab	–	Tofacitinib	–	–	–
60–99	Telaprevir	–	–	–	–	–	–
100–149	–	Telaprevir	–	–	–	–	–
150–200	Pasireotide	Pertuzumab	–	–	–	Tofacitinib	–
201–896	Ipilimumab, Vandetanib	Ipilimumab, Vandetanib	Ipilimumab, Taliglucerase alfa	–	–	Pasireotide, Pertuzumab	–

^a^ Multiple of average monthly per capita income.

Note: Information about income is available in Table 1.

Three products in Argentina and Chile – and two products in Brazil – cost less than the average monthly per capita income (Table 5). Only one product (rivaroxavan) cost less than the average monthly per capita income in all countries, but four products cost more than 100 times this amount. The results using monthly household disposable income and monthly household wealth were very similar to findings for average monthly per capita income (details available from the corresponding author).

**Table 5:** Cost of a course of treatment (or a year of treatment for chronic conditions) for new products tested in Latin America in relation to average monthly per capita income, 2014

Cost^a^	Argentina	Brazil	Chile	Colombia	Ecuador	Mexico	Peru
< 1	Indacaterol, Linagliptin, Rivaroxavan	Rivaroxavan, Roflumilast	Apixaban, Indacaterol, Rivaroxavan	Rivaroxavan	–	Rivaroxavan	Rivaroxavan
1–4	Apixaban, Ticagrelor	Apixaban, Ticagrelor	–	Apixaban, Indacaterol	Indacaterol	Apixaban, Azilsartan, Linagliptin, Ticagrelor	–
5–9	–	Belatacept	Aflibercept, Belimumab	–	–	–	–
10–19	Regorafenib	Aflibercept, Belimumab	–	Aflibercept	–	–	–
20–39	Aflibercept, Belatacept, Tofacitinib	–	–	Tofacitinib	–	Tofacitinib	–
40–59	Telaprevir	–	–	–	–	–	–
60–99	–	Ipilimumab, Pertuzumab, Telaprevir	Ipilimumab	–	–	Pertuzumab	–
100–149	Ipilimumab, Pasireotide	Pasireotide	–	–	–	–	–
150–200	Vandetanib	Vandetanib	–	–	–	–	–
201–203	–	–	Taliglucerase alfa	–	–	–	–

^a^ Multiple of average monthly per capita income.

Note: Information about income is available in Table 1.

The bulletins of Prescrire and/or the Health Research Group evaluated 25 of the 32 products and determined that 20 (80%) had no advantage over existing treatments and had significant side-effects. According to these sources, and other bulletins reviewed by Prescrire, the remaining five products (crizotinib, enzalutamide, ipilimumab, pasireotide, telaprevir) may provide some therapeutic benefit to a subset of patients, but the risk–benefit ratio was uncertain (details available from the corresponding author).

## Discussion

According to our data, 20 months after the FDA had approved the commercialization of 33 products in the USA, only eight (25%) had been registered and commercialized in all the Latin American countries where they had been tested. Thirty percent (10/33) had been neither registered nor commercialized in any of the countries; 45% (15/33) were registered and some of these were commercialized in several countries.

The suboptimal implementation of differential or tiered pricing for pharmaceuticals, a strategy recommended by WHO to enhance access to pharmaceuticals,^22^ has led to arbitrary decisions. For example, the antiretroviral drug atazanavir is supplied for free to people living with human immunodeficiency virus in Peru. The country pays about 6.34 United States dollars (US$) per tablet while Argentina pays US$ 3.04, Brazil US$ 1.00 and the Plurinational State of Bolivia US$ 0.48 (personal communication), prices that with the exception of the Plurinational State of Bolivia do not correspond to the wealth of the countries. It is conceivable that the establishment of national or public health sector affordability thresholds would render new pharmaceuticals accessible.

Within pharmaceutical companies, there seems to be little communication between the research and development units and those responsible for marketing the final products. Clinical trials are outsourced when the countries meet the conditions of the sponsor or of the contract research organizations managing the trial, such as having large urban centres with researchers willing to enrol patients, expedited approval of protocols and sufficient patients who can be readily recruited.^23^ The registration and marketing of new products are business decisions based on a country’s regulatory conditions, the presence of a business affiliate or partner, the willingness of the health system to include a product in its formulary, the number of patients who can afford the treatment and estimates of drug profitability for the company.

Latin American agencies responsible for the approval of clinical trials, including the research ethics committees, do not consider if the tested products will be available and affordable. Only Brazil requires that all drugs tested in the country be registered when found to be safe and effective. Resolution 446 of its regulatory agency (Anvisa) reads: “When developing new drugs, if safety and effectiveness is proven, its registration is obligatory in Brazil.” However, as our findings demonstrate, it appears that the regulation is not being enforced.

In sum, neither the sponsors of the clinical trials, nor the regulatory agencies, nor any of the bodies that approved ethical declarations regarding clinical trials have suggested pre-trial mechanisms to ensure that new products will be available and affordable in those countries where testing has taken place. Without such mechanisms, declarations such as those of the Council for International Organizations of Medical Sciences (CIOMS), the Universal Declaration on Bioethics and Human Rights, or the Declaration of Helsinki are difficult to fulfil (Box 2). International Ethical Guidelines for Biomedical Research Involving Human Subjects state that:

Box 2 **International ethical guidelines asserting that post trial access to treatment should be ensured**

**Council for International Organizations of Medical Sciences^24^**

**Guideline 10: Research in populations and communities with limited resources**

Before undertaking research in a population or community with limited resources, the sponsor and the investigator must make every effort to ensure that:

- the research is responsive to the health needs and the priorities of the population or community in which it is to be carried out; and
- any intervention or product developed, or knowledge generated, will be made reasonably available for the benefit of that population or community.

**Commentary on Guideline 10**

This is applicable especially to research conducted in countries where governments lack the resources to make such products or benefits widely available. Even when a product to be tested in a particular country is much cheaper than the standard treatment in some other countries, the government or individuals in that country may still be unable to afford it. If the knowledge gained from the research in such a country is used primarily for the benefit of populations that can afford the tested product, the research may rightly be characterized as exploitative and, therefore, unethical.

The negotiation should cover the health-care infrastructure required for safe and rational use of the intervention, the likelihood of authorization for distribution and decisions regarding payments, royalties, subsidies, technology and intellectual property, as well as distribution costs, when this economic information is not proprietary.

In general, if there is good reason to believe that a product developed or knowledge generated by research is unlikely to be reasonably available to, or applied to the benefit of, the population of a proposed host country or community after the conclusion of the research, it is unethical to conduct the research in that country or community.

**Universal Declaration on Bioethics and Human Rights. Article 15 – Sharing of benefits^25^**

Benefits resulting from any scientific research and its applications should be shared with society as a whole and within the international community, in particular with developing countries.

**Declaration of Helsinki (2013)^26^**

Item 22. In clinical trials, the protocol must also describe appropriate arrangements for post-trial provisions.

“In general, if there is good reason to believe that a product developed or knowledge generated by research is unlikely to be reasonably available to, or applied to the benefit of the population of a proposed host country or community after the conclusion of the research, it is unethical to conduct the research in the country or community.”(Commentary on Guideline 10).^27^

Latin American regulatory agencies have very limited resources for assessing the safety and efficacy of new products, some of which are very complex. For this reason, they depend on the FDA or the European Medicines Agency’s decisions to grant approval. The pharmaceutical industry claims that conducting trials in Latin America strengthens research capacity in biomedical science,^23^ but it could also be suggested that high payments to principal investigators lure some of them away from developing other products needed in the region, such as treatments for dengue, malaria and leishmaniasis or from the development of generic biophamaceuticals, a move that could save lives and money.^28^^,^^29^ Industry prices are not related to product development cost^30^^,^^31^ and some new products are unaffordable even in high-income countries. In both the USA and the United Kingdom of Great Britain and Northern Ireland,^32^^–^^35^ physicians and health authorities are increasingly reluctant to accept expensive medications offering little improvement over existing cheaper alternatives with demonstrated safety and efficacy.

In January 2014, a new consensus framework for ethical collaboration between patients’ organizations, health-care professionals and the pharmaceutical industry was published.^36^ The framework states that:

“Continuing to advocate and support the principle that all human subject research must have legitimate scientific purpose, aims to improve health outcomes and be ethically conducted…”^36^

To comply with this consensus and with universally-accepted ethical principles,^27^^,^^37^^,^^38^ the pharmaceutical industry should: (i) reconsider research and commercial strategies to ensure that new products add therapeutic value to the existing therapeutic arsenal at an affordable price; (ii) include the estimated local prices for potential new products in clinical trial protocols so that the regulatory agencies and research ethics committees can consider the affordability of the product when authorizing the research (iii)  work with regulatory agencies in the countries where products are tested to ensure registration and availability of new products that prove to be safe and effective.

### Study limitations

The information on registration and commercialization status of new products may contain inaccuracies. We identified and corrected some errors through triangulation, but others may not have been detected. Some FDA reviews did not specify which of the clinical trials were pivotal. Even though we also gathered information from trial sponsors, we may have included trials that technically might not be considered pivotal. There are unavoidable limitations in estimating thresholds of affordability, since financial sacrifices and risks cannot be easily defined by others. These are personal decisions that are influenced by personal values and culture.

Evaluating the price of drugs continues to be complex and currently there is no standard method. Despite their shortcomings, country observatories, which tend to be based on the WHO/HAI methods, are probably the best and most reliable sources of information. Currency variations add to the complexity of reporting pricing information across countries. We priced the drugs in September 2014, but the data used to determine monthly income is from 2013. Moreover, in the Latin American countries included in this study, income is very poorly distributed. If we were to remove the highest two income deciles, the income per capita for the rest of the population would be drastically reduced, and therefore the affordability threshold would have to be lowered.

## Conclusion

Many pharmaceutical products tested in Latin America are unavailable and/or unaffordable to most of the population and add little therapeutic value compared to existing treatments. There is an urgent need to determine the public-sector affordability thresholds for new pharmaceutical products, and efforts should be made to ensure that ethics committees can take into consideration the affordability and clinical relevance of the new products in their assessment of the clinical trial protocols. The products included in this study did not respond to the most pressing medical needs of people in the region and may have diverted scientific resources from addressing issues of higher relevance. While governments welcome the investments that accompany foreign trials, it is important to document their opportunity costs.
