# Corrigendum

**DOI:** 10.2471/BLT.16.100516

**Published:** 2016-05-02

In Volume 93, Issue 10, October 2015, throughout pages 674−83 “Rivaroxavan” should read: “Rivaroxaban”.

